# Antibody Response After a Second Dose of the BNT162b2 mRNA COVID-19 Vaccine in Liver Transplant Recipients

**DOI:** 10.3389/ti.2022.10321

**Published:** 2022-03-16

**Authors:** Akiyoshi Sakai, Tetsuji Morishita, Hidetoshi Matsunami

**Affiliations:** ^1^ Department of Clinical Laboratory, Matsunami General Hospital, Gifu, Japan; ^2^ Department of Internal Medicine, Matsunami General Hospital, Gifu, Japan; ^3^ Matsunami Research Park, Gifu, Japan; ^4^ Matsunami General Hospital, Gifu, Japan

**Keywords:** severe acute respiratory syndrome coronavirus 2, antibody responses, immunodeppressants, mycophenolate mofetil, coronavirus disease 2019

Dear Editors,

Coronavirus disease 2019 (COVID-19) caused by severe acute respiratory syndrome coronavirus 2 (SARS-CoV-2) has spread globally, and the World Health Organization declared a pandemic on March 11, 2020. A widespread health emergency with social and economic disruptions remains in effect worldwide. Vaccination against SARS-CoV-2 is therefore an essential tool to control the global COVID-19 pandemic.

Pioneering studies on vaccines against SARS-CoV-2 have verified their safety and efficacy in general populations (1). However, data remain scarce regarding fragile populations such as organ transplant recipients (2–4). Indeed, individuals on immunosuppressants have been specifically excluded from SARS-CoV-2 vaccine trials (1). Previous studies have described suppressed antibody titers in patients receiving mycophenolate mofetil (5). Thus, in consideration of the potential for blunted immune responses to vaccinations, quantification of the immunogenicity of SARS-CoV-2 vaccines in fragile populations represents an urgent issue.

We aimed to evaluate antibody response after the second dose of BNT162b2 mRNA vaccine (Pfizer/BioNTech). We measured IgG antibody titers to the S receptor-binding domain (RBD) in liver transplant recipients and healthy controls who had received two doses of BNT162b2 mRNA vaccine. We also analyzed how immunosuppressant regimens affected antibody responses.

We included individuals who had received the second dose of BNT162b2 mRNA COVID-19 vaccine between March and August 2021. This study included 56 liver transplant patients and 42 healthy controls at Matsunami General Hospital. Blood was collected at least 14 days after the second vaccination. RBD-IgG titers were measured using the SARS-CoV-2 S-IgG (IC) Assay Reagent assay kit (Fujirebio Inc. Tokyo, Japan). Titers greater than 1.0 arbitrary units (AU)/mL were considered positive (detection range, 0.1–20 AU/mL). This study conforms to the principles outlined in the 1975 Declaration of Helsinki and its later amendments. The study protocol was approved by the Ethics Committee of Matsunami General Hospital (approval no. 498, 2021).

RBD-IgG antibody titers were measured in 56 liver transplant recipients. The median age of liver transplant recipients was 65.0 years, comprising 76.8% males (*n* = 43) and 23.2% females (*n* = 13). None of participants had a prior polymerase chain reaction–confirmed diagnosis of COVID-19, hospitalization and death between March, 2021 and September, 2021. Liver transplant recipients showed significantly decreased antibody titers as compared with healthy controls (Figure 1A). Liver transplant recipients developed significantly lower antibody titers when compared with healthy controls (adjusted mean difference −7.42; 95% confidence interval, −12.81 to −2.03; *p* = 0.008). Median time between liver transplantation and BNT162b2 vaccination was 15.5 years. Calcineurin inhibitor-based immunosuppressive therapy was used in 91.1% (*n* = 51), mycophenolate mofetil in 58.9% (*n* = 33), steroids in 1.8% (*n* = 1), and mTOR inhibitors in 1.8% (*n* = 1) among liver transplant recipients.

**FIGURE 1:**
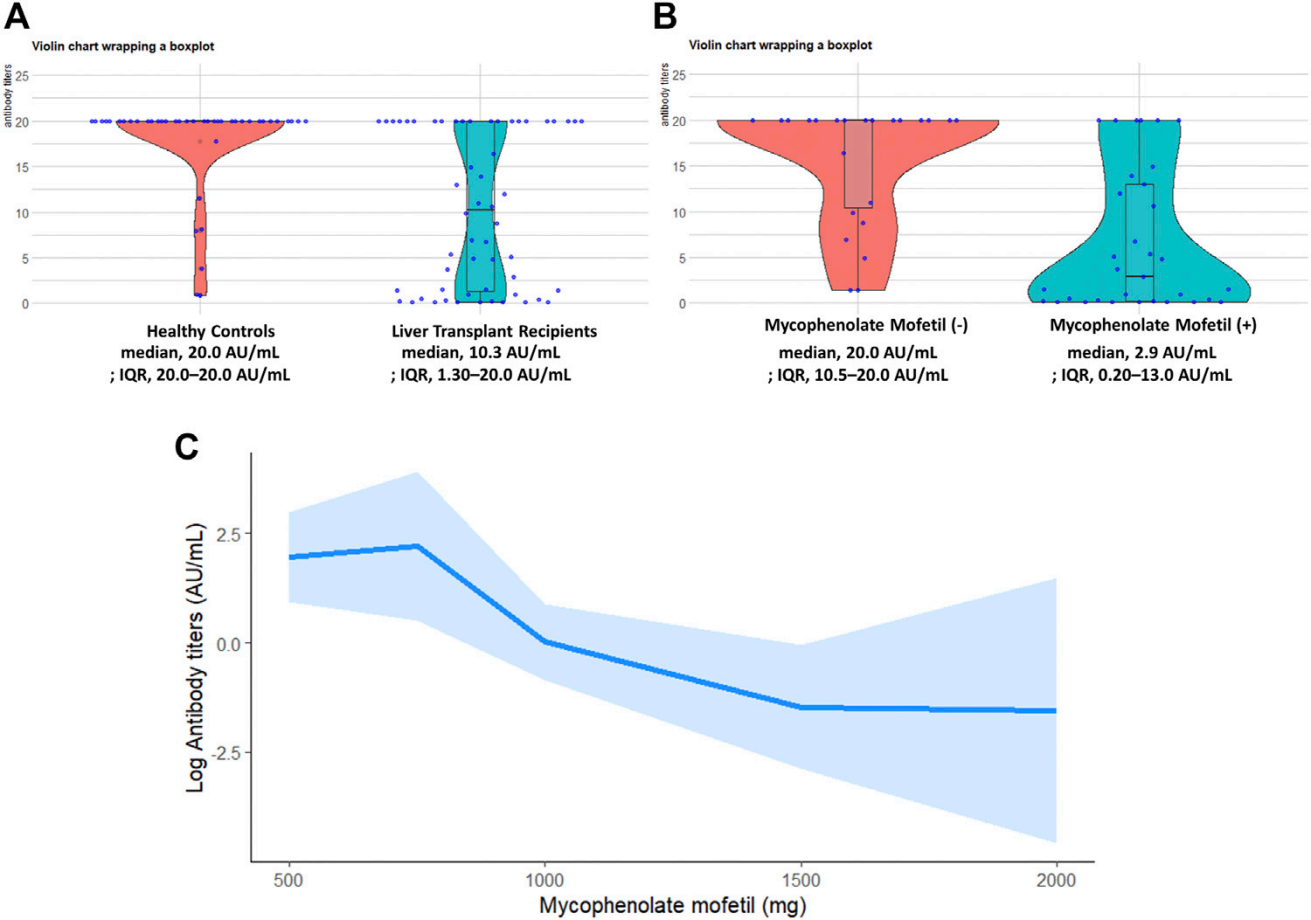
**(A)** Violin charts wrapping a box plot show RBD-IgG antibody levels in the liver transplant recipient group (*n* = 56) and healthy donor group (*n* = 42) after the second dose. **(B)** Violin charts wrapping a box plot show RBD-IgG antibody levels in recipients taking mycophenolate mofetil (*n* = 33) and recipients not taking mycophenolate mofetil (*n* = 23) after the second dose of vaccine. Each point represents an individual patient, and horizontal lines indicate medians. Values above the detection limit are plotted as 20 AU/mL. Values below the detection limit are plotted as 0.1 AU/mL. **(C)** Association between RBD-IgG antibody titers and mycophenolate mofetil dose in liver transplant recipients by restricted cubic spline model with four knots. The shaded area represents the 95% confidence interval. IQR, interquartile range; RBD, S receptor-binding domain.

The overall seroconversion rate after the second vaccination was 86.7% in study participants. Liver transplant recipients showed a lower seroconversion rate (44/56; 78.6%) than healthy controls (41/42; 97.6%). The seroconversion rate was lower in recipients taking mycophenolate mofetil (21/33, 63.6%) than in those not taking mycophenolate mofetil (23/23, 100%; *p* = 0.001), whereas the seroconversion rate was higher in recipients taking calcineurin inhibitor (42/51, 82.4%) than in those not taking calcineurin inhibitor (2/5, 40.0%; *p* = 0.06).

Figure 1B compares antibody titers between liver transplant recipients with or without use of mycophenolate mofetil. Development of RBD IgG antibody titers was less likely in liver transplant recipients taking mycophenolate mofetil (median, 2.9 AU/mL; IQR, 0.20–13.0 AU/mL) than in those not taking mycophenolate mofetil (median, 20.0 AU/mL; IQR, 10.5–20.0 AU/mL; *p* < 0.001).

A restricted cubic spline plot (Figure 1C) shows the relationship between RBD IgG antibody titers and total mycophenolate mofetil dose in liver transplant recipients. An inverse linear relationship between RBD IgG antibody titers and mycophenolate mofetil dose was detected (*p* for effect = 0.008, *p* for non-linearity = 0.24).

Our findings in liver transplant recipients confirmed suboptimal immunogenicity after the second dose of BNT162b2 vaccine, supporting findings from other studies of kidney transplant recipients (3), allogeneic hematopoietic stem-cell transplant recipients (6) and lung transplant recipients (7). Concern remains about the occurrence of severe COVID-19 in some vaccinated immunocompromised transplant recipients.

The seroconversion rate was as low as 63.6% (21/33) in patients taking mycophenolate mofetil. Even in patients with confirmed seroconversion, antibody levels were low, suggesting that the threshold for protective immunity had not been reached. Restricted cubic spline modeling also verified a linear dose-response relationship between mycophenolate mofetil dose and reduced antibody titers in our study. Reduced antibody responses among organ transplant recipients, including liver transplant recipients, suggest that these recipients may remain at high risk of COVID-19 even after the second and third doses of mRNA vaccine.

Our findings have clinical implications for liver transplant recipients, emphasizing the need to consider a fourth dose of vaccination and the assessment of antibody titers even after the third dose of vaccination. Although a third vaccine dose was well tolerated by solid organ transplant recipients who had an insufficient antibody response after two dose of vaccination, the serological response was heterogeneous and a large proportion of recipients remain at risk for COVID-19 (8, 9). Approaches to improve antibody responses in transplant recipients may require temporary reduction or withdrawal of mycophenolate mofetil, or replacement to other immunosuppressants and additional measures such as subsequent a fourth dose of vaccination (10). The assessment of antibody titers even after the third dose of vaccination might be important to discriminate patients who should maintain their barrier measures.

Longevity of the antibody titer and the optimal period of monitoring antibody titers are still unveiled. Further longitudinal studies are warranted to investigate how cellular immune responses will be maintained or whether waning antibody titers still provide protection from breakthrough infections in transplant recipients with larger participants.

In conclusion, we have revealed mycophenolate mofetil contributed the attenuated antibody acquisition against SARS-CoV-2 among liver transplant recipients with dose-dependent manner. For populations that prove unlikely to acquire antibodies, active measurement of RBD-IgG antibodies may be warranted to assess protective immunity against COVID-19 and the need for additional vaccination.

## Data Availability

The datasets generated and/or analyzed during the present study are not publicly available due to ethical/privacy reasons but are available from the corresponding author on reasonable request.

## Abbreviations

COVID-19, coronavirus disease 2019; RBD, S receptor-binding domain; SARS-CoV-2, severe acute respiratory syndrome coronavirus 2.
